# Exploring the Rheological Properties of 3D Bioprinted Alginate-Based Hydrogels for Tissue Engineering

**DOI:** 10.3390/biomimetics10080491

**Published:** 2025-07-24

**Authors:** R. Palacín-García, L. Goñi, T. Gómez-del Río

**Affiliations:** Durability and Mechanical Integrity of Structural Materials, Rey Juan Carlos University, Tulipán s/n, 28933 Madrid, Spain

**Keywords:** bioink, bioprinting alginate/polyacrylamide hydrogels, viscoelasticity, rheological properties

## Abstract

The development of alginate/polyacrylamide hydrogels for various biomedical applications has attracted significant interest, particularly due to their potential use in wound healing and tissue engineering. This study explores the fabrication of these hydrogels via 3D bioprinting with ultraviolet light curing, focusing on how the alginate concentration and curing speed impact their mechanical properties. Rheological testing was employed to examine the viscoelastic behavior of alginate/polyacrylamide hydrogels manufactured using a 3D bioprinting technique. The relaxation behavior and dynamic response of these hydrogels were analyzed under torsional stress, with relaxation curves fitted using a two-term Prony series. Fourier Transform Infrared (FTIR) spectroscopy was also employed to assess biocompatibility and the conversion of acrylamide. This study successfully demonstrated the printability of alginate/polyacrylamide hydrogels with varying alginate contents. The rheological results indicated that 3D bioprinted hydrogels exhibited significantly high stiffness, viscoelasticity, and long relaxation times. The curing speed had a minimal impact on these properties. Additionally, the FTIR analysis confirmed the complete conversion of polyacrylamide, ensuring no harmful effects in biological applications. The study concludes that 3D bioprinting significantly enhances the mechanical properties of alginate/polyacrylamide hydrogels, with the alginate concentration playing a key role in the shear modulus. These hydrogels show promising potential for biocompatible applications such as wound healing dressings.

## 1. Introduction

Tissue engineering is the application of engineering and life sciences to the development of artificial substitutes to restore the function of damaged tissues and organs [1]. Recent advancements in this field have led to the experimental production of certain organs, such as a synthetic bladder [2]. The organ shortage is an escalating health issue due to the increasing demand and limited supply of organ donors [3]. Therefore, the availability of artificial organs may mitigate the organ shortage in the future. The complexity of manufacturing artificial skin is relatively low compared to other more intricate tissues and organs [4]. However, some challenges remain unresolved. The development of elements such as the vasculature and innervation, integration with the native tissue, and the controlled distribution of multiple cell types and biomaterials within a single construct are among the ongoing challenges in tissue engineering [5,6].

The structure of the skin comprises three layers that fulfill distinct functions. The skin thickness varies depending on the species, sex, age, and body region. The epidermis is a hard, keratinized, almost impermeable, 50–150 μm wide layer in humans that prevents water loss and the ingress of external substances [7,8].The dermis, which is 0.15–4 mm thick in humans and is mainly composed of collagen fibers, is the major layer responsible for the mechanical stability of the skin [7]. The hypodermis, primarily composed of adipose tissue, is the deepest layer, providing insulation and shock absorption [7,9].

A wound is a major health concern due to complications in diabetes patients and infections [10]. Certain bioactive materials can improve wound healing by reducing inflammation and promoting the growth of fibroblasts, keratinocytes, and other cells involved in skin repair [11,12]. Specifically, alginates reduce wound pain, lower the infection risk, decrease odor, and assist in hemostasis. Alginates also maintain a moist environment and absorb exudates [13]. Therefore, they are the main ingredient in several commercial wound care products [10]. However, there is currently no single commercially available artificial skin that can fully replicate all skin layers [14]. Although a few attempts have been made to fabricate three-layer skin constructs, more research in hypodermal engineering is required [15]. In addition, most artificial skin constructs lack complex features such as pigmentation, sweat glands, and hair follicles [16].

Wound treatment is not the only application of artificial skin. To substitute animal testing, alternative methods for evaluating skin corrosion or irritation by chemical products are under development. Several methods use reconstructed human epidermis models [17]. In the European Union, the ban on animal testing for cosmetic products has promoted the development of artificial skin models for testing skin products [18]. Hydrogels are also used as vectors for controlled drug delivery [19].

Hydrogels are tridimensional networks of polymer chains filled with water. Due to their physical and structural similarities to natural tissues, some hydrogels are highly biocompatible, making them suitable materials for tissue engineering. Hydrogels contain hydrophilic functional groups, which provide impressive water absorption. Certain hydrogel matrices promote cellular adhesion and growth. Hydrogels composed of natural materials, such as alginate, chitosan, and gelatin, have good biological properties, including cell encapsulation and biocompatibility [20]. Hydrogels composed of synthetic materials, such as polyacrylamide, poly(viniylpyrrolidone), and poly(lactic acid), usually have better mechanical properties than natural hydrogels [21].

Additive manufacturing techniques facilitate precise spatial control during fabrication. Among these, 3D bioprinting is one of the most prevalent methods. Bioprinting is the adaptation of additive manufacturing for the fabrication of biomaterials, enabling the creation of complex shapes that mimic the form of real organs. The layer-by-layer deposition of the material enables the encapsulation of multiple cell types and accurate control over the internal construct geometry [22]. There are several bioprinting technologies available, including inkjet printing, laser-assisted printing, and extrusion printing. Extrusion printing is an appealing option due to its affordability and capability to print multiple inks simultaneously [6,20]. However, this technology requires the use of inks with specific properties to obtain proper shape fidelity. The ability to form and maintain reproducible 3D forms using a specific bioprinting technique is defined as printability. Bioinks suitable for extrusion should exhibit shear-thinning behavior, so that the fluid becomes less viscous as it is extruded from the syringe, forming a filament [23]. Consequently, the availability of materials for hydrogel production by 3D bioprinting is reduced compared to bulk fabrication [24,25]. There are three dispensing mechanisms available for extrusion bioprinting: pneumatic-, piston-, and screw-based. In pneumatic-based bioprinters, pressurized air (or nitrogen) pushes the bioink out of the nozzle, with air pressure controlling the amount of extruded material. Piston and screw systems apply a higher force on the bioink and provide more direct control over the extrusion rate [26].

The gel-forming mechanism is also critically important for 3D bioprinting. Each layer of material must acquire enough structural integrity before the following layer is deposited, but gelation within the printer feed system must be avoided [27]. Therefore, precise control of the gelation time is required. Chemically cross-linked hydrogels, such as polyacrylamide, can be printed by adding a photoinitiator and exposing the printing surface to light. Most non photo-curable hydrogels can be chemically modified to become photo-curable [24]. That is the case of gelatin methacryloyl (GelMA), a gelatin modified with photo-cross-linkable methacrylamide groups [28]. Whereas some bioprinting techniques, such as direct light processing (DLP) and stereolithography (SLA), are restricted to light-induced gelation, extrusion printing is also compatible with other methods. For instance, polymers formed through physical interactions, such as gelatin, can be solidified by a drop in temperature [24].

Alginate is a biodegradable, non-toxic, and non-immunogenic polysaccharide [29]. It is usually obtained from brown algae, making it a natural, low-cost, and eco-friendly material. It has a considerable amount of carboxyl and hydrogel groups, which facilitate adhesion to biological tissues through hydrogen bond interactions [30]. Alginate solutions gel fast when mixed with multivalent cations such as Ca^2+^, a mechanism that can be used for bioprinting [31]. Aqueous solutions of alginate are shear-thinning and easy to prepare and extrude, making it one of the most used components in the design and fabrication of bioinks [31].

Alginates form a soft and ductile yet mechanically weak network due to the weak and reversible alginate–metal bonds responsible for cross-linking. By interpenetrating with a stiff and rigid but brittle network, such as polyacrylamide, a double network (DN) hydrogel is formed. DN hydrogels exhibit mechanical properties that are orders of magnitude greater than their discrete components [32,33]. When a force is applied, some bonds within the polyacrylamide network are broken, dissipating energy, while the alginate–metal network distributes the remaining tension over a large region of the hydrogel [34].

Polyacrylamide is among the most popular materials for hydrogel manufacturing. Acrylamide is a cheap synthetic monomer that produces tougher hydrogels than most natural hydrogels. Their stiffness can be customized by altering the concentrations of the cross-linker and monomer. Their transparent nature allows for easy visual inspection. Additionally, polymerization can be easily initiated using a photoinitiator and ultraviolet light [35]. The most used cross-linker for acrylamide polymerization is N,N′-methylenebisacrylamide.

Three-dimensional printing techniques alter the mechanical properties of the resulting hydrogel. In particular, the bond strength between layers may be lower than interactions within a layer, generating anisotropic solids. The mechanical response of hydrogels produced by molding and casting are well known [36]. However, mechanical characterization studies of extruded constructs are usually limited to the elastic behavior, and neither the mechanism nor time-dependent mechanical behavior resulting from the bioprinting process are considered [37]. Furthermore, the intrinsic characteristics of 3D printed materials, such as anisotropy, requires the usage of additional characterization techniques [38].

Proper mechanical properties are essential for all biomaterials. The material must resist the mechanical stresses encountered during activities performed by the patient. In addition, mechanical stiffness similar to that of native tissues is necessary for cell growth, improving the regeneration of the damaged zone [39]. Hydrogels are poroviscoelastic materials. The viscoelastic behavior results from conformational changes in the polymer structure [40], while the poroelastic behavior is caused by the flow of solvent through the pores of the hydrogel [41]. Rheology is the primary method to study the viscoelastic properties of hydrogels [42]. Most experiments are conducted within the linear viscoelastic region of the material, and so the results are independent of the amount of the amount of applied strain or stress [43]. Some rheology experiments, such as torsion, do not cause solvent displacement in the hydrogel, as the sample volume is maintained. Therefore, no poroelastic behavior is observed, allowing the viscoelastic behavior to be studied independently [44].

The viscoelastic behavior of hydrogels results from conformational changes within the polymeric structure, aiming to reach an equilibrium state [26,40,45]. One of the most used tests to characterize viscoelastic behavior is the relaxation test, where a fixed displacement or deformation is applied to a hydrogel. The load decreases during the test while the displacement is maintained. This delayed response is independent of the characteristic size of the test carried out, as long as it is much smaller than the characteristic dimension of the polymeric structure [44,45,46,47,48]. The viscoelastic behavior of hydrogels can be described using a standard viscoelastic solid model or the Maxwell–Wierchert model [22,23,24,49]. The relaxation function, represented as *G(t)*, can be fitted to a Prony series using Equation (1):

(1) Gt=G∞+∑i=1nGi·e−tτi

where $G_{\infty }$ is the value after the viscoelastic process is relaxed, and $G_{i}$ and *τ_i_* are the elastic modulus and the relaxation time associated with each of the elements of the Maxwell—Wiechert model.

In this work, the suitability of extrusion 3D bioprinting for alginate–acrylamide mixtures with different concentrations of alginate was studied. Then, hydrogels from those solutions were fabricated using extrusion 3D printing. The mechanical properties of the obtained hydrogels were studied using a hybrid rheometer. Lastly, to ensure that the hydrogels did not have harmful remaining amounts of acrylamide, we analyzed their chemical composition by FTIR spectroscopy. All these studies aim to test the optimal use of polyacrylamide–alginate hydrogels for biomedical applications, specifically for use in wound dressings for skin injuries.

## 2. Materials and Methods

### 2.1. Preparation of the Hydrogels

**Figure 1:**
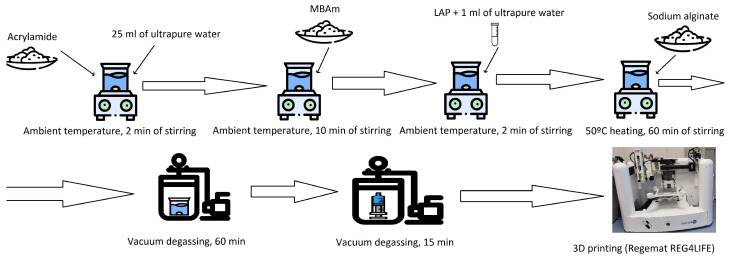
Fabrication procedure for 3D printing with a hydrogel bioink. MBAm: N,N′-methylenebisacrylamide. LAP: lithium phenyl-2,4,6-trimethylbenzoylphosphinate. Icons are from flaticon.com, used under license.

Hydrogels composed of alginate and polyacrylamide were prepared using a two-step approach based on the method outlined by R. Bai et al. [50]. In the first step, covalent cross-linking of the acrylamide chains was achieved, while in the second step, ionic cross-linking of the alginate chains occurred. For the covalent cross-linking process, UV light exposure for 3D bioprinting applications was used. The process for preparing the bioink is illustrated in Figure 1. A detailed list of the ingredients used in each hydrogel formulation can be found in Table 1.

For the covalent cross-linking step, a 1.98 M solution of acrylamide (synthesis grade, Merck Group) was prepared in ultrapure water (18.2 MΩ·cm^−1^ at 25 °C, from a Millipore Direct-Q 3 system). To this solution, sodium alginate (ACS grade, BoroGlass S.L.) was added at ratios of 1:4.5 and 1:6 by weight relative to acrylamide. After complete dissolution, the cross-linking agent for the polyacrylamide network, N, N′-methylenebisacrylamide (≥99.5% purity, Merck Group), was introduced at a composition of 15% T and 0.6% C, following the equations described by Chirani et al. [51] and Denisin et al. [52]. The resulting precursor solution was degassed for 60 min under a vacuum of approximately 50 mm Hg to remove air bubbles that may have formed during mixing.

(2) $$\%T\;\left(\frac{w}{v}\right)=\frac{weight\;of\;monomer\;\left(g\right)+weight\;of\;x-crosslinker\;\left(g\right)}{total\;volume\;\left(mL\right)}$$

(3) $$\%C\;\left(\frac{w}{v}\right)=\frac{weight\;of\;x-crosslinker\;\left(g\right)}{weight\;of\;monomer\;\left(g\right)+weight\;of\;x-crosslinker\;\left(g\right)}$$

The bioinks were printed using the Regemat REG4LIFE extrusion 3D printer. The printing temperature was maintained at 23 °C using a temperature-controlled syringe module, which is close to room temperature and facilitates the immediate cross-linking of acrylamide, enabling the formation of a 3D structure. For these samples, 0.01 g of lithium phenyl-2,4,6-trimethylbenzoylphosphinate (LAP) (≥95% purity, Sigma-Aldrich, Saint Louis, MO, USA) was added as a photoinitiator to the precursor solution, corresponding to 0.0028 times the weight of acrylamide. After thorough mixing, the solution was loaded into an opaque syringe with a 0.41 mm tip diameter. The printing speed was set at 20 mm/s, and the printed layers had a thickness of 0.15 mm. After each layer was deposited, a UV lamp emitting light at 365 nm was used for curing, with scanning speeds ranging between 2 and 10 mm/s. The UV lamp was positioned 7 mm from the sample, delivering an irradiance intensity of 14,000 mW/cm^2^. Finally, the samples were punched with an 8 mm diameter punch.

For ionic cross-linking, a 0.5 M solution of high-purity calcium sulfate (Phampur, Ph Eur, BP, NF, BoroGlass. S.L., ref. CA02840500) was prepared in Milli-Q water. The samples were immersed in this solution for 24 h, allowing the Ca^2+^ ions to diffuse into the hydrogel and induce ionic cross-linking of the alginate. To remove any excess salt, the samples were thoroughly washed with ultrapure water and stored for 24 h before testing.

### 2.2. Characterization of the Bioinks

To produce accurate and functional 3D hydrogels, bioinks with suitable viscosity, good recovery, and proper viscoelastic properties are essential. These characteristics must be considered when designing bioinks for specific tissue engineering applications [53,54]. During the extrusion process in printing, the bioink must become less viscous under shear stress and return to its initial viscosity once the stress is removed, such as when it is deposited onto the printing bed. Therefore, the bioink needs to have strong thixotropic properties and exhibit shear-thinning behavior, which is dependent on the process.

The rheological properties of the inks play a crucial role in extrusion-based 3D printing of hydrogels because the material undergoes various external forces and recovery stages during the printing process, which significantly influence its properties and performance. A rheological study helps to determine how easily the material flows through the nozzle and its ability to retain its shape after extrusion. To assess the thixotropic properties and recoverability of the inks, a three-step test was performed:

- Initial stage—A low shear rate (approximately 0.011 s^−1^) was applied for 120 s, simulating the hydrogel’s initial state before printing.
- High shear rate stage—The shear rate was significantly increased to around 100 s^−1^, disrupting the internal gel structure and temporarily lowering the viscosity for 120 s. This step simulates the behavior of the hydrogel during extrusion.
- Recovery stage—The shear rate was reduced to a low value for another 120 s, allowing the bioink to regain stability and return to its original viscosity, which simulates the final state of the hydrogel after it has been printed.

Different compositions were initially tested in the 3D bioprinting process. However, not all combinations proved suitable for producing 3D bioprinted hydrogel samples with well-defined shapes. If the hydrogel was in optimal condition, the extruded material would exhibit a proper morphology, with a smooth surface and consistent width across all dimensions, resulting in uniform shapes. In contrast, when the hydrogel was under-gelated, the extruded filament would appear more liquid-like, causing the lower layers to spread out on the printing surface. To assess the ability of the hydrogels to be printed with accurate shapes, two parameters were evaluated: the circularity and printability of the mixtures.

The circularity (*C*) of a closed area is calculated using the following formula:

(4) $$C=\frac{4\pi A}{L^{2}}$$

where *L* represents the perimeter and *A* represents the area. A perfect circle has a circularity value of 1, with the value decreasing as the shape deviates from a circle. For example, the circularity of a square is equal to π/4. Ouyang et al. [55] and Bom et al. [56] defined the printability (*P*) of a bioink based on the circularity of square-shaped printed structures using the following equation:

(5) $$P=\frac{\pi }{4}\times \frac{1}{C}=\frac{L^{2}}{16A}$$

Under ideal 3D printing conditions, the printed shape would be a perfect square, and the printability value (*P*) would be 1. To evaluate the shape fidelity during the 3D printing process, optical images of a single printed layer were captured and analyzed using IC Measure software version 4.0 (The Imaging Source) for each alginate concentration.

### 2.3. Characterization of the Hydrogels

#### 2.3.1. Fourier Transform Infrared (FTIR) Spectroscopy

FTIR spectroscopy was utilized to investigate the physical and chemical interactions in 3D bioprinted hydrogels. The analysis was conducted using a Nicolet iS50 Thermo Fisher Scientific FT-IR spectrophotometer (Waltham, MA, USA). Each sample was measured with 64 scans averaged to obtain clear spectra, and the resolution was set to 4 cm^−1^. The spectral range covered was from 4000 cm^−1^ to 400 cm^−1^, ensuring comprehensive data acquisition. This technique allowed the identification of functional groups and structural changes in the hydrogel samples, providing valuable insights into the material′s characteristics after the printing process.

To perform the FTIR analysis, it was necessary to dry the hydrogels and grind them in a mortar to increase the exposed superficial area, thereby achieving a spectrum with clear vibration peaks of the polyacrylamide and alginate bonds. In hydrated hydrogels, the broadening effects caused by water molecule vibrations hindered the identification of the polymer peaks, as they overlapped with the relevant spectral features.

#### 2.3.2. Rheology

The storage modulus (G′) and loss modulus (G″) of polyalginate/polyacrylamidenate hydrogel formulations were determined using a Discovery HR-10 Waters TA Instruments rheometer with a parallel plate setup. To prepare the samples, hydrogel disks with an 8 mm diameter and 5 mm thickness were fabricated and allowed to swell in an isotonic solution for a minimum of 24 h. To prevent slipping during testing, sandpaper was placed between the sample and the device′s disks.

The samples were submerged in water, and the measurement was conducted at a temperature of 25 °C. An axial force ranging from 0.02 to 0.05 N was applied to compress the samples, ensuring proper contact between the plates and the sample. Relaxation and frequency sweep tests were carried out to evaluate the viscoelastic properties.

Each test was divided into three stages:

- Stage 1—A twist of 10^−8^ rad was applied, followed by a 900 s relaxation period. This stage was intended for stabilization, and no data were collected for characterization.
- Stage 2—A twist of 0.02 rad was applied, followed by a 1200 s relaxation period, with data being recorded every 0.2 s.
- Stage 3—A dynamic frequency sweep was conducted under strain-controlled conditions, ranging from 0.01 Hz to the maximum frequency allowed by the test conditions, with a maximum strain of 1%. Data were collected at five points per decade.

At least five samples were tested for each hydrogel formulation to ensure statistical reliability. The maximum frequency in hybrid rheometers depends on the material’s stiffness and the equipment’s inertial moment. This value was determined for each hydrogel, and the maximum angular velocity was set at 10 rad/s for the 3D bioprinted hydrogels. Table 2 summarizes the main parameters of the rheological tests conducted.

The stress relaxation behavior of each hydrogel was analyzed using data from Stage 2. The hydrogel formulations were modeled as linear viscoelastic materials. The long-term elastic modulus, $G_{\infty }$, was determined by averaging the fully relaxed data collected during the final seconds of the relaxation period. These properties of the material were used in the optimization of the viscoelastic material model, as detailed below.

For making comparative evaluations of different materials, a two-term Prony series was used to model the relaxation data based on linear viscoelasticity. The time-dependent relaxation function, G(t), was described by the following equation:

(6) Gt=G∞+G∞−G0A·e−tτ1+1−A·e−tτ2

where $G_{\infty }\;$ is the long-term elastic shear modulus, $G_{0}$ is the instant modulus, $\tau_{1}$ and $\tau_{2}$ are relaxation times, and *A* is a dimensionless pre-exponential factor between 0 and 1.

## 3. Results and Discussion

### 3.1. Properties of the Bioinks

**Figure 2:**
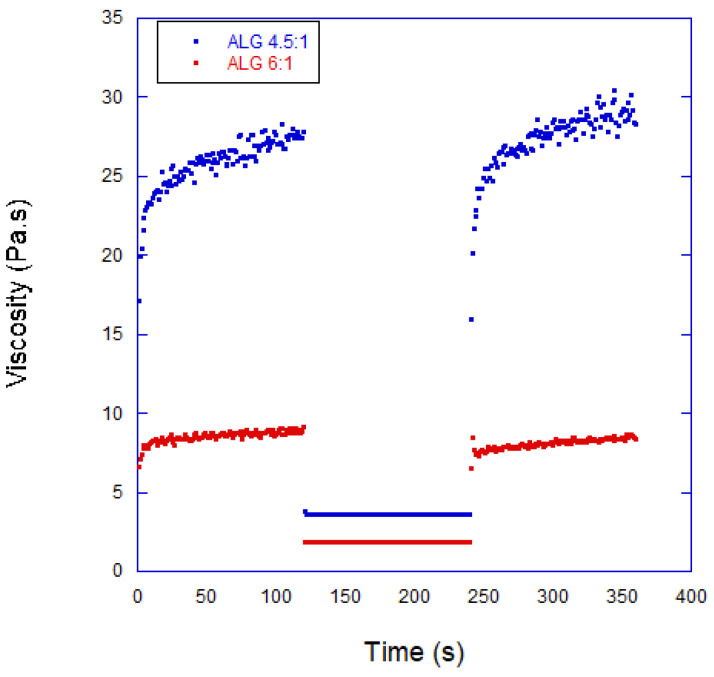
Analysis of the thixotropic properties of the bioink for the 1:6 alginate/polyacrylamide composition. The shear rate applied during the first and third stages was 100 s^−1^, while during the second stage, it was 0.011 s^−1^.

Figure 2 illustrates the thixotropic behavior of the inks, showcasing the viscosity recovery of the ink with a 1:6 composition. Initially, the viscosity was measured at 7.2 Pa.s, but at a shear rate of 100 s^−1^, it decreased to 1.5 Pa.s. Once the shear rate was reduced, the viscosity recovered about 75% of its initial value within 30 s, and after a longer period (100 s), the recovery reached 87%. These favorable thixotropic properties led to the selection of these hydrogels for bioprinting applications.

To assess the suitability of the bioinks for bioprinting, recovery tests were conducted using 40 mm diameter parallel plates with sandpaper surfaces, and a 1 mm gap was set between the plates. The results, presented in Figure 2, revealed that the bioinks exhibited predominantly elastic behavior, with nearly instantaneous recovery and a slight increase in viscosity when the high shear rate was applied. Additionally, the bioinks displayed a strong shear-thinning effect, as well as rheopectic behavior characterized by a rapid viscosity increase followed by a slower one after the shear was applied.

**Figure 3:**
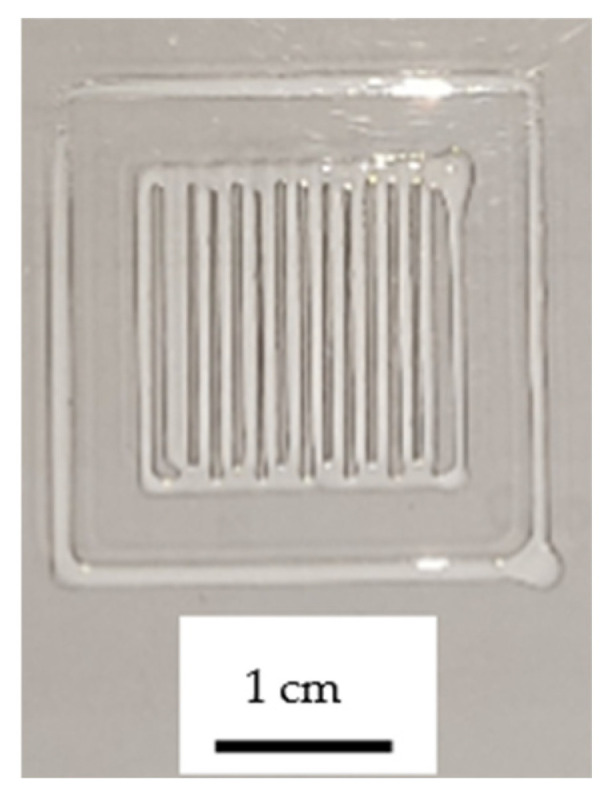
One of the pictures used for measuring printability.

The printability of the bioinks was evaluated by printing a single layer of constructs and capturing images for analysis. The printed images were processed using IC Measure software, and the printability factor was determined using Equation (5). Three samples were printed for each bioink composition. To ensure statistical reliability, a confidence interval was calculated using Equation (7):

(7) $$\overset{-}{x}\pm (\frac{t*s}{\sqrt{n}})$$

where $\overset{-}{x}$ is the average printability value, *t* represents the corresponding Student’s t-distribution value for two tails at 95% confidence, ss is the standard deviation of the sample, and *n* denotes the number of samples. Since only a single layer was printed, the UV light curing time was considered non-relevant, and the measurements were taken at a lamp speed of 5 mm/s. The printed design was a square with 10 mm sides and a 1 mm separation between lines. One of the printed hydrogels is shown in Figure 3.

The printability results obtained were (1.04 ± 0.03) for the alginate/polyacrylamide 1:6 bioink and (1.00 ± 0.01) for the 1:4.5 composition bioink. Both compositions demonstrated good printability, and although there were minor differences, the printability of the polyadrylamide/alginate 1:4.5 bioink showed a smaller confidence interval.

### 3.2. FTIR Evaluation of the Hydrogels

**Figure 4:**
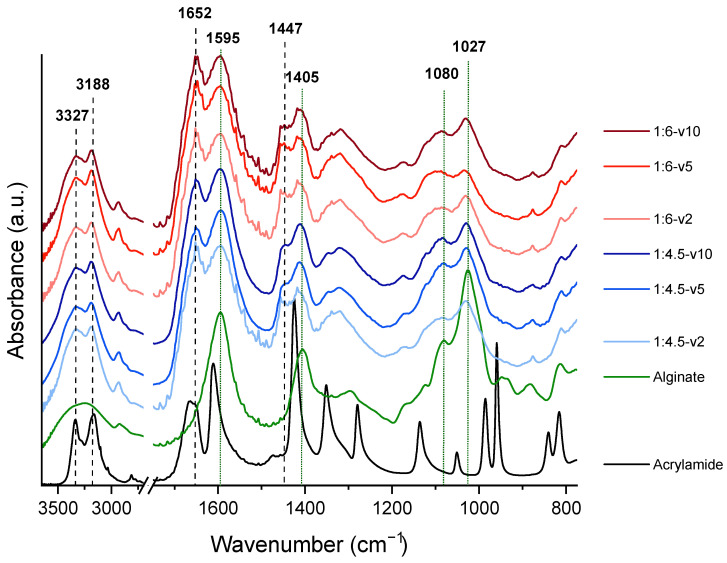
FTIR spectra of the polyacrylamide–alginate 3D bioprinted hydrogels.

Figure 4 presents the FTIR spectra of the hydrogels, as well as those of the acrylamide and alginate used in their synthesis. In the alginate spectrum, a broad band centered around 3245 cm^−1^ was observed, corresponding to the stretching vibration of O–H groups.

Characteristic bands at 1595 cm^−1^ and 1405 cm^−1^, attributed to the asymmetric and symmetric stretching vibrations of the carboxylate groups, respectively, were also observed. Additionally, bands at 1080 cm^−1^ and 1027 cm^−1^, corresponding to C–C–C and C–O–C stretching vibrations in the polysaccharide backbone, were identified. These bands were present in the hydrogel spectra, albeit with variations in intensity. To quantify these changes, the peak at 1652 cm^−1^, corresponding to the C=O stretching vibration of the carboxamide groups in polyacrylamide, was used as a reference due to its consistent intensity across all hydrogel samples.

The intensity ratios of the alginate bands relative to the 1652 cm^−1^ peak showed differences depending on the alginate concentration. For instance, in the 1:4.5 polyacrylamide–alginate hydrogels (18% *w/w*), the ratio of r_1595/1652_ was 1.05, while in the 1:6 polyacrylamide–alginate hydrogels (14% *w/w*), it was 0.99. These variations suggest that the intensity of the carboxylate bands increases with a higher alginate content. Similarly, the ratios of the 1405 cm^−1^ and 1027 cm^−1^ bands to the 1652 cm^−1^ peak also increased with the alginate concentration, with the most pronounced change observed in the 1027 cm^−1^ peak (see Table 3).

Regarding polyacrylamide in the hydrogel spectra, the broad O–H stretching band observed in the alginate spectrum split into two peaks at 3327 cm^−1^ and 3188 cm^−1^, corresponding to the asymmetric and symmetric stretching vibrations of the –NH_2_ (carboxamide) groups in polyacrylamide, respectively. The peak at 1447 cm^−1^, corresponding to CH_2_ bending in the polymer backbone, remained present and stable across the hydrogel samples, providing further evidence of the incorporation of polyacrylamide into the network. Regardless of the curing rate, no characteristic bands of unpolymerized acrylamide were detected in any of the hydrogels. The absence of C=C stretching vibrations characteristic of unpolymerized acrylamide, typically observed at 1610 cm^−1^ (C=C bond) and 995–900 cm^−1^ (vinyl-type C=C stretching), confirms the complete polymerization of the monomer. Instead, the presence of the 1652 cm^−1^ peak further supports the successful formation of polyacrylamide and its cross-linking, ensuring that no residual acrylamide monomer remains in the hydrogel.

The FTIR analysis verifies the successful formation of polyacrylamide–alginate hydrogels and the absence of unpolymerized acrylamide, confirming the non-toxicity of the final material.

### 3.3. Viscoelastic Properties of the Hydrogels

**Figure 5:**
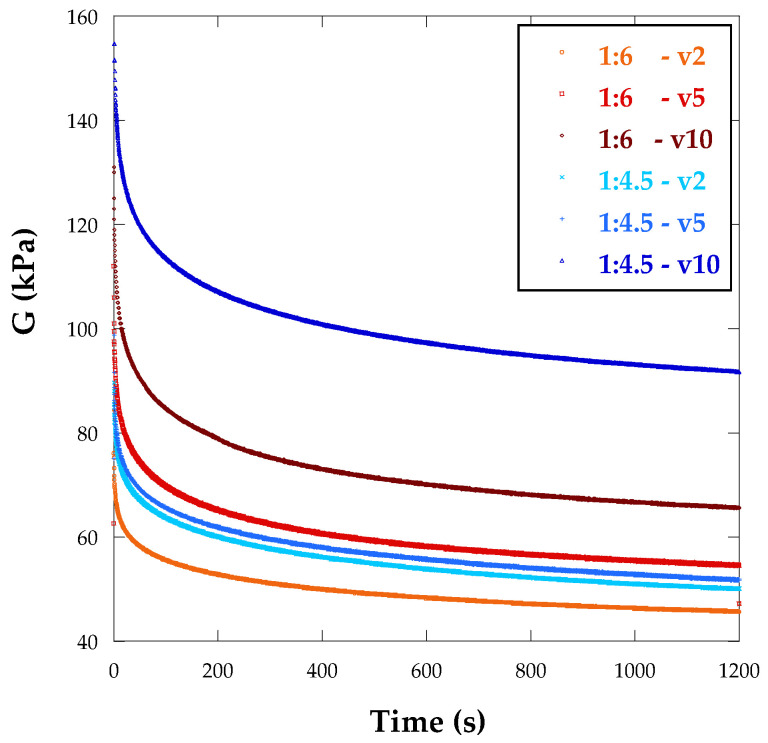
Average curves of the experimental relaxation of the shear modulus versus time curves for different alginate/polyacrylamide hydrogel samples. The samples included different alginate contents and UV lamp velocities for the 3D bioprinted samples: 10, 5, and 2 mm/s.

Figure 5 displays the average loading curves and relaxation curves measured for the 3D bioprinted alginate/polyacrylamide hydrogels at a 0.02 rad rotation angle. Some degree of variation was observed in these relaxation curves and, consequently, average curves were calculated for each hydrogel condition based on the alginate percentage and curing lamp speed. It was evident that the stiffness of the material was significantly influenced by the alginate concentration. Specifically, increasing the alginate concentration resulted in a higher shear modulus, regardless of the UV lamp speed in the 3D bioprinting methodology. Furthermore, the stiffness of the 3D bioprinted hydrogels was notably higher compared to those fabricated through molding, regardless of the UV lamp speed [41].

During the relaxation process, equilibrium is achieved when the load value becomes constant. It appears that 3D bioprinted samples exhibit long relaxation times, both for short- and long-term relaxation. To fully understand this relationship, more detailed analysis of the experimental curves is required.

The average curves presented in Figure 5 can be attributed to the relaxation phenomena in the hydrogels, which result from their viscoelastic behavior. The viscoelastic characteristics of the hydrogels were modeled using a two-term Prony series, as described in previous research [22,23,49]. Equation (3) was applied to fit the relaxation curves with two exponential terms.

The parameters resulting from fitting the relaxation curves are presented in Table 4. The findings indicate that the 3D printed samples exhibit higher shear modulus values, with relaxation times also being longer in these samples, signifying a more viscous behavior.

Additionally, dynamic oscillatory frequency sweep experiments were conducted to analyze the viscoelastic properties of the various hydrogels. These tests were performed under controlled conditions at a temperature of 25.0 ± 0.1 °C using a constant angular strain amplitude of 1%. The angular frequency varied between 0.01 and 10 rad/s. Higher frequencies were avoided due to potential inertial effects on the device that could interfere with the measured torsional momentum.

A comparison of the storage modulus (G’) and loss modulus (G”) revealed that there was no crossover point between these curves, indicating that the cross-linking of alginate in the hybrid hydrogels was a permanent, chemically cross-linked process. The storage modulus (G’) consistently exceeded the loss modulus (G”) across the entire frequency range, demonstrating the predominance of the elastic behavior in these hydrogels and indicating minimal viscous flow. This type of response is commonly observed in gels, whether they are protein-based [35,57] or polysaccharide-based hydrogels [57,58].

The hybrid hydrogels displayed similar characteristics to chemically cross-linked hydrogels, with G’ and G” values that were largely independent of frequency. The main difference between them was that chemically cross-linked hydrogels exhibited a higher loss modulus, suggesting less viscous flow. When the storage modulus exceeds the loss modulus, it signals a solid-like structure for the hydrogel [59,60]. Moreover, the absence of a crossover point between the storage and loss moduli across the tested frequencies further confirms that the hydrogels have entangled fibrous networks.

In a similar study, Jamburidze et al. [61] used frequency sweep tests to compare agarose hydrogels with biological tissues. They found that the G’/G” ratio of the tested hydrogels was 10, a value that aligns closely with the properties observed in naturally occurring tissues, suggesting that these hydrogels could be suitable for applications in tissue engineering.

## 4. Conclusions

In this study, the printability of alginate/polyacrylamide hydrogels was successfully demonstrated through the extrusion-based 3D printing process. The viscoelastic behavior of alginate/polyacrylamide hydrogels produced using 3D printing was analyzed through rheological testing to explore how these processes impact the relaxation behavior. Additionally, different concentrations of alginate were examined for each manufacturing technique. The following key conclusions were drawn from the experimental results:

- Alginate/polyacrylamide hydrogels with varying alginate contents were successfully fabricated using 3D printing with photopolymerization, and their printability was evaluated.
- Rheological tests proved to be effective in analyzing the viscoelastic behavior of alginate/polyacrylamide hydrogels. Since torsional tests mainly involve pure shear stress, they allowed for the study of the viscoelastic behavior without interference from other phenomena, such as poroelasticity.
- The relaxation curves of the hydrogels were modeled using a two-term Prony series equation, providing a comprehensive understanding of their viscoelastic characteristics.
- The manufacturing process significantly impacts the mechanical properties of alginate/polyacrylamide hydrogels in two main ways. First, 3D printing notably enhances the stiffness of the hydrogels. Second, both viscoelasticity and relaxation times are increased in hydrogels produced via 3D printing. However, the velocity of the UV lamp used for curing does not seem to influence these behaviors to a significant extent.
- Differences were observed among hydrogels produced with the same method but differing cross-linker concentrations. For 3D printed hydrogels, the shear modulus increased as the alginate content decreased.
- The experimental results indicated that the cross-linking of alginate in these hydrogels was irreversible. The elastic component dominated across all hydrogels and frequencies studied, confirming that the hydrogels primarily exhibited elastic behavior.

## Figures and Tables

**Table 1:** Ingredients for the two hydrogel compositions. Cross-linker: N,N′-methylenebisacrylamide. LAP: lithium phenyl-2,4,6-trimethylbenzoylphosphinate.

Composition of Alginate/Acrylamide	Acrylamide (g)	Cross-Linker (g)	Sodium Alginate (g)	LAP (g)
1:4.5	3.525	0.0225	0.7833	0.01
1:6	3.525	0.0225	0.5875	0.01

**Table 2:** Experimental data for rheological tests.

Sample Name	Polyacrylamide–Alginate Proportion	UV Lamp Velocity (m/s)	Frequency Range (Hz)	Holding Time (s)
1:4.5 v2	1:4.5	2	0.01–100	1200
1:4.5 v5	1:4.5	5	0.01–100	1200
1:4.5 v10	1:4.5	10	0.01–100	1200
1:6 v2	1:6	2	0.01–100	1200
1:6 v5	1:6	5	0.01–100	1200
1:6 v10	1:6	10	0.01–100	1200

**Table 3:** Calculations of the height ratios from the polyacrylamide–alginate hydrogel FTIR spectra.

**Composition**	**UV Lamp** **Velocity**	**Ratio with C=O Stretching [1652 cm^−1^] of Polyacrylamide**		
		**COO Asymmetric [1595 cm^−1^]**	**COO Symmetric [1405 cm^−1^]**	**C-O-C [1027 cm^−1^]**
1:4.5	v2	0.98	0.76	0.67
	v5	1.10	0.78	0.88
	v10	1.06	0.74	0.76
1:6	v2	0.97	0.72	0.64
	v5	1.00	0.73	0.54
	v10	1.00	0.72	0.67
**Composition**	**UV Lamp** **Velocity**	**Ratio with CH_2_ Bending [1447 cm^−1^] of Polyacrylamide**		
		**COO Asymmetric [1595 cm^−1^]**	**COO Symmetric [1405 cm^−1^]**	**C-O-C [1027 cm^−1^]**
1:4.5	v2	1.43	1.10	1.00
	v5	1.65	1.18	1.32
	v10	1.69	1.17	1.20
1:6	v2	1.47	1.10	1.00
	v5	1.45	1.05	0.78
	v10	1.58	1.14	1.06

**Table 4:** Fitting parameters obtained from the average relaxation curve of alginate/polyacrylamide hydrogels based on the two-term Prony series Equation (3). $G_{\infty }$ is the asymptotic transverse elastic modulus value when the sample is fully relaxed and $G_{0}$ is the instantaneous load measured at the initial step of the relaxation.

Polyacrylamide–Alginate Proportion	Manufacturing Process	Fitting Parameters				
		Short Relaxation Time (s)	Long Relaxation Time (s)	Pre-Exponential Factor, A (-)	$\mathrm{Instant}\;\mathrm{Shear}\;\mathrm{Modulus}\;G_{0}$ (kPa)	$\mathrm{Shear}\;\mathrm{Modulus}\;G_{\infty }$ (kPa)
1:4.5	3D printing/v10	21	380	0.51	149	91
1:4.5	3D printing/v5	26	390	0.50	91	52
1:4.5	3D printing/v2	29	460	0.44	80	49
1:6	3D printing/v10	24	340	0.49	114	65
1:6	3D printing/v5	25	363	0.50	92	54
1:6	3D printing/v2	27	430	0.46	68	45

## Data Availability

Raw/processed data are available upon reasonable request addressed to the corresponding author.

## References

[1] Fox C.F., Skalak R. (1988). Tissue Engineering: Proceedings of a Workshop, Held at Granlibakken, Lake Tahoe, California, February 26–29, 1988.

[2] Oberpenning F., Meng J., Yoo J.J., Atala A. (1999). De novo reconstitution of a functional mammalian urinary bladder by tissue engineering. Nat. Biotechnol..

[3] Chidume T. (2021). Promoting older adult fall prevention education and awareness in a community setting: A nurse-led intervention. Appl. Nurs. Res..

[4] Ng W.L., Wang S., Yeong W.Y., Naing M.W. (2016). Skin Bioprinting: Impending Reality or Fantasy?. Trends Biotechnol..

[5] Zhang B., Gao L., Ma L., Luo Y., Yang H., Cui Z. (2019). 3D Bioprinting: A Novel Avenue for Manufacturing Tissues and Organs. Engineering.

[6] Fang Y., Guo Y., Liu T., Xu R., Mao S., Mo X., Zhang T., Ouyang L., Xiong Z., Sun W. (2022). Advances in 3D Bioprinting. Chin. J. Mech. Eng. Addit. Manuf. Front..

[7] Jor J.W.Y., Parker M.D., Taberner A.J., Nash M.P., Nielsen P.M.F. (2013). Computational and experimental characterization of skin mechanics: Identifying current challenges and future directions. WIREs Mech. Dis..

[8] Huzaira M., Rius F., Rajadhyaksha M., Anderson R.R., González S. (2001). Topographic Variations in Normal Skin, as Viewed by In Vivo Reflectance Confocal Microscopy. J. Investig. Dermatol..

[9] Wilkes G.L., Brown I.A., Wildnauer R.H. (1973). The biomechanical properties of skin. CRC Crit. Rev. Bioeng..

[10] Sahana T.G., Rekha P.D. (2018). Biopolymers: Applications in wound healing and skin tissue engineering. Mol. Bio. Rep..

[11] Eming S.A., Krieg T., Davidson J.M. (2007). Inflammation in Wound Repair: Molecular and Cellular Mechanisms. J. Investig. Dermatol..

[12] Stramer B.M., Mori R., Martin P. (2007). The Inflammation–Fibrosis Link? A Jekyll and Hyde Role for Blood Cells during Wound Repair. J. Investig. Dermatol..

[13] Enoch S., Grey J.E., Harding K.G. (2006). Non-surgical and drug treatments. BMJ.

[14] Chua A.W.C., Khoo Y.C., Tan B.K., Tan K.C., Foo C.L., Chong S.J. (2016). Skin tissue engineering advances in severe burns: Review and therapeutic applications. Burn. Trauma.

[15] Kaur A., Midha S., Giri S., Mohanty S. (2019). Functional Skin Grafts: Where Biomaterials Meet Stem Cells. Stem Cells Int..

[16] Ng W.L., Qi J.T.Z., Yeong W.Y., Naing M.W. (2018). Proof-of-concept: 3D bioprinting of pigmented human skin constructs. Biofabrication.

[17] Liu Y., Li N., Chen L., Alépée N., Cai Z. (2020). A ready-to-use integrated in vitro skin corrosion and irritation testing strategy using EpiSkinTM model in China. Toxicol. In Vitro.

[18] Géniès C., Jacques-Jamin C., Duplan H., Rothe H., Ellison C., Cubberley R., Schepky A., Lange D., Klaric M., Hewitt N.J. (2020). Comparison of the metabolism of 10 cosmetics-relevant chemicals in EpiSkinTM S9 subcellular fractions and in vitro human skin explants. J. Appl. Toxicol..

[19] Li J., Mooney D.J. (2016). Designing hydrogels for controlled drug delivery. Nat. Rev. Mater..

[20] Krishna D.V., Sankar M.R. (2023). Extrusion based bioprinting of alginate based multicomponent hydrogels for tissue regeneration applications: State of the art. Mater. Today Commun..

[21] Mehta P., Sharma M., Devi M. (2023). Hydrogels: An overview of its classifications, properties, and applications. J. Mech. Behav. Biomed. Mater..

[22] Ersumo N., Witherel C.E., Spiller K.L. (2016). Differences in time-dependent mechanical properties between extruded and molded hydrogels. Biofabrication.

[23] Naghieh S., Chen X. (2021). Printability–A key issue in extrusion-based bioprinting. J. Pharm. Anal..

[24] Kirchmajer D.M., Gorkin R., Panhuis M.I.H. (2015). An overview of the suitability of hydrogel-forming polymers for extrusion-based 3D-printing. J. Mater. Chem. B.

[25] Jungst T., Smolan W., Schacht K., Scheibel T., Groll J. (2016). Strategies and Molecular Design Criteria for 3D Printable Hydrogels. Chem. Rev..

[26] Chen D.X.B. (2019). Extrusion Bioprinting of Scaffolds for Tissue Engineering Applications.

[27] Bakarich S.E., Panhuis M.I.H., Beirne S., Wallace G.G., Spinks G.M. (2013). Extrusion printing of ionic–covalent entanglement hydrogels with high toughness. J. Mater. Chem. B.

[28] Gao Q., Niu X., Shao L., Zhou L., Lin Z., Sun A., Fu J., Chen Z., Hu J., Liu Y. (2019). 3D printing of complex GelMA-based scaffolds with nanoclay. Biofabrication.

[29] Pawar S.N., Edgar K.J. (2012). Alginate derivatization: A review of chemistry, properties and applications. Biomaterials.

[30] Ren P., Yang L., Wei D., Liang M., Xu L., Zhang T., Hu W., Zhang Z., Zhang Q. (2023). Alginate/polyacrylamide host-guest supramolecular hydrogels with enhanced adhesion. Int. J. Biol. Macromol..

[31] Axpe E., Oyen M. (2016). Applications of Alginate-Based Bioinks in 3D Bioprinting. Int. J. Mol. Sci..

[32] Pragya A., Mutalik S., Younas M.W., Pang S.-K., So P.-K., Wang F., Zheng Z., Noor N. (2021). Dynamic cross-linking of an alginate–acrylamide tough hydrogel system: Time-resolved in situ mapping of gel self-assembly. RSC Adv..

[33] Li J., Illeperuma W.R.K., Suo Z., Vlassak J.J. (2014). Hybrid Hydrogels with Extremely High Stiffness and Toughness. ACS Macro Lett..

[34] Sun J.-Y., Zhao X., Illeperuma W.R.K., Chaudhuri O., Oh K.H., Mooney D.J., Vlassak J.J., Suo Z. (2012). Highly stretchable and tough hydrogels. Nature.

[35] Subramani R., Izquierdo-Alvarez A., Bhattacharya P., Meerts M., Moldenaers P., Ramon H., Van Oosterwyck H. (2020). The Influence of Swelling on Elastic Properties of Polyacrylamide Hydrogels. Front. Mater..

[36] Christensen K., Davis B., Jin Y., Huang Y. (2018). Effects of printing-induced interfaces on localized strain within 3D printed hydrogel structures. Mater. Sci. Eng. C.

[37] Zhang T., Yan K.C., Ouyang L., Sun W. (2013). Mechanical characterization of bioprinted in vitro soft tissue models. Biofabrication.

[38] Farzadi A., Solati-Hashjin M., Asadi-Eydivand M., Osman N.A.A. (2014). Effect of Layer Thickness and Printing Orientation on Mechanical Properties and Dimensional Accuracy of 3D Printed Porous Samples for Bone Tissue Engineering. PLoS ONE.

[39] Engler A.J., Sen S., Sweeney H.L., Discher D.E. (2006). Matrix Elasticity Directs Stem Cell Lineage Specification. Cell.

[40] Caccavo D., Cascone S., Lamberti G., Barba A.A. (2018). Hydrogels: Experimental characterization and mathematical modelling of their mechanical and diffusive behaviour. Chem. Soc. Rev..

[41] Reinhards C., Rico A., Rodríguez J. (2021). Crosslinker concentration effect on the poroviscoelastic relaxation of polyacrylamide hydrogels using depth-sensing indentation. Polym. Test..

[42] Yan C., Pochan D.J. (2010). Rheological properties of peptide-based hydrogels for biomedical and other applications. Chem. Soc. Rev..

[43] Stieger M. (2002). The Rheology Handbook—For users of rotational and oscillatory rheometers. Appl. Rheol..

[44] Caccavo D., Lamberti G. (2017). PoroViscoElastic model to describe hydrogels’ behavior. Mater. Sci. Eng. C.

[45] Cacopardo L., Guazzelli N., Nossa R., Mattei G., Ahluwalia A. (2019). Engineering hydrogel viscoelasticity. J. Mech. Behav. Biomed. Mater..

[46] Hu Y., Suo Z. (2012). Viscoelasticity and poroelasticity in elastomeric gels. Acta Mech. Solida Sin..

[47] Fitzgerald M.M., Bootsma K., Berberich J.A., Sparks J.L. (2015). Tunable Stress Relaxation Behavior of an Alginate-Polyacrylamide Hydrogel: Comparison with Muscle Tissue. Biomacromolecules.

[48] Strange D.G.T., Fletcher T.L., Tonsomboon K., Brawn H., Zhao X., Oyen M.L. (2013). Separating poroviscoelastic deformation mechanisms in hydrogels. Appl. Phys. Lett..

[49] Wang Q.-M., Mohan A.C., Oyen M.L., Zhao X.-H. (2014). Separating viscoelasticity and poroelasticity of gels with different length and time scales. Acta Mech. Sin..

[50] Bai R., Yang Q., Tang J., Morelle X.P., Vlassak J., Suo Z. (2017). Fatigue fracture of tough hydrogels. Extrem. Mech. Lett..

[51] Chirani N., L’Hocine Y., Lukas G., Federico L.M., Soumia C., Silvia F. (2015). History and Applications of Hydrogels. J. Biomed. Sci..

[52] Denisin A.K., Pruitt B.L. (2016). Tuning the Range of Polyacrylamide Gel Stiffness for Mechanobiology Applications. ACS Appl. Mater. Interfaces.

[53] Schwab A., Levato R., D’Este M., Piluso S., Eglin D., Malda J. (2020). Printability and Shape Fidelity of Bioinks in 3D Bioprinting. Chem. Rev..

[54] Zhang Z., Jin Y., Yin J., Xu C., Xiong R., Christensen K., Ringeisen B.R., Chrisey D.B., Huang Y. (2018). Evaluation of bioink printability for bioprinting applications. Appl. Phys. Rev..

[55] Ouyang L., Yao R., Zhao Y., Sun W. (2016). Effect of bioink properties on printability and cell viability for 3D bioprinting of embryonic stem cells. Biofabrication.

[56] Bom S., Ribeiro R., Ribeiro H.M., Santos C., Marto J. (2022). On the progress of hydrogel-based 3D printing: Correlating rheological properties with printing behaviour. Int. J. Pharm..

[57] Pettinelli N., Sabando C., Rodríguez-Llamazares S., Bouza R., Castaño J., Valverde J.C., Rubilar R., Frizzo M., Recio-Sánchez G. (2024). Sodium alginate-g-polyacrylamide hydrogel for water retention and plant growth promotion in water-deficient soils. Ind. Crops Prod..

[58] Daemi H., Barikani M. (2012). Synthesis and characterization of calcium alginate nanoparticles, sodium homopoly-mannuronate salt and its calcium nanoparticles. Sci. Iran..

[59] Alizadeh Behbahani B., Jooyandeh H., Taki M., Falah F. (2024). Evaluation of the probiotic, anti-bacterial, anti-biofilm, and safety properties of *Lacticaseibacillus paracasei* B31-2. LWT.

[60] Ying X., Wang H., Liu J., Li X. (2018). Polyacrylamide-grafted calcium alginate microspheres as protein-imprinting materials. Polym. Bull..

[61] Jamburidze A., De Corato M., Huerre A., Pommella A., Garbin V. (2017). High-frequency linear rheology of hydrogels probed by ultrasound-driven microbubble dynamics. Soft Matter.

